# Pyogenic granuloma of the lower labial mucosa during pregnancy: a case report

**DOI:** 10.3389/fsurg.2026.1913013

**Published:** 2026-08-19

**Authors:** Heng Pan, Jiashan Li, Zhouhuan Chen

**Affiliations:** Department of Stomatology, Maternity & Child Healthcare Hospital of Longgang District, [Affiliated Shenzhen Women and Children’s Hospital (Longgang) of Shantou University Medical College], ShenZhen, China

**Keywords:** case report, gestation, lobular capillary hemangioma, lower labial mucosa, pyogenic granuloma

## Abstract

Extragingival pyogenic granuloma (PG) of the lower labial mucosa is rare during pregnancy, with only a few cases previously reported. This report describes the management of a PG on the lower labial mucosa in a 26-year-old patient at 18 weeks of gestation, representing the earliest reported gestational presentation requiring surgical intervention. The patient presented with a progressively enlarging mass and recurrent bleeding. Given these clinical indications and the lack of established safety data for non-surgical treatments during pregnancy, surgical excision was performed under local anesthesia using lidocaine without epinephrine rather than deferring treatment until after delivery. Histopathology confirmed lobular capillary hemangioma with surface ulceration. At 3-month follow-up, no recurrence was observed, and both maternal and fetal conditions remained stable. This case demonstrates that surgical excision during the second trimester under local anesthesia is feasible and safe, and provides a perioperative management reference for clinicians facing similar cases.

## Introduction

Pyogenic granuloma (PG) is a common benign vascular proliferative lesion of the oral cavity (1). The term “pyogenic granuloma” is a clinical designation, while “lobular capillary hemangioma” is the preferred histopathological term. Hormonal changes during pregnancy can induce or aggravate this condition, leading to the term “gestational pyogenic granuloma.” Most reported cases of gestational PG occur on the gingiva. In contrast, cases involving the labial mucosa are rarely reported, and their clinical features and perioperative management lack specific discussion. This report presents a case of PG on the lower labial mucosa in a patient at 18 weeks of gestation. We discuss its pathogenesis, differential diagnosis, and perioperative management to improve the clinical understanding of extragingival PG during pregnancy.

## Case report

### Patient timeline

Approximately 3 months before presentation: onset of a small red spot on the lower labial mucosa. Gradual enlargement over the following 3 months, with recurrent bleeding during eating and speaking. At 18 weeks of gestation: presentation to our department and surgical excision. Two weeks postoperatively: suture removal; wound healed well. Three months postoperatively: follow-up; no recurrence; prenatal check-ups normal.

A 26-year-old female patient at 18 weeks of gestation presented with a mass on the lower labial mucosa for over 3 months, which had progressively enlarged and was prone to ulceration and bleeding after friction during eating or speaking. The patient reported no significant pain, fever, or numbness. She had no history of local chronic irritation (such as residual roots or poor restorations), no bad oral habits (such as lip biting), and no history of hematologic diseases. Routine prenatal check-ups were normal, and she had no other systemic comorbidities.

**Figure 1:**
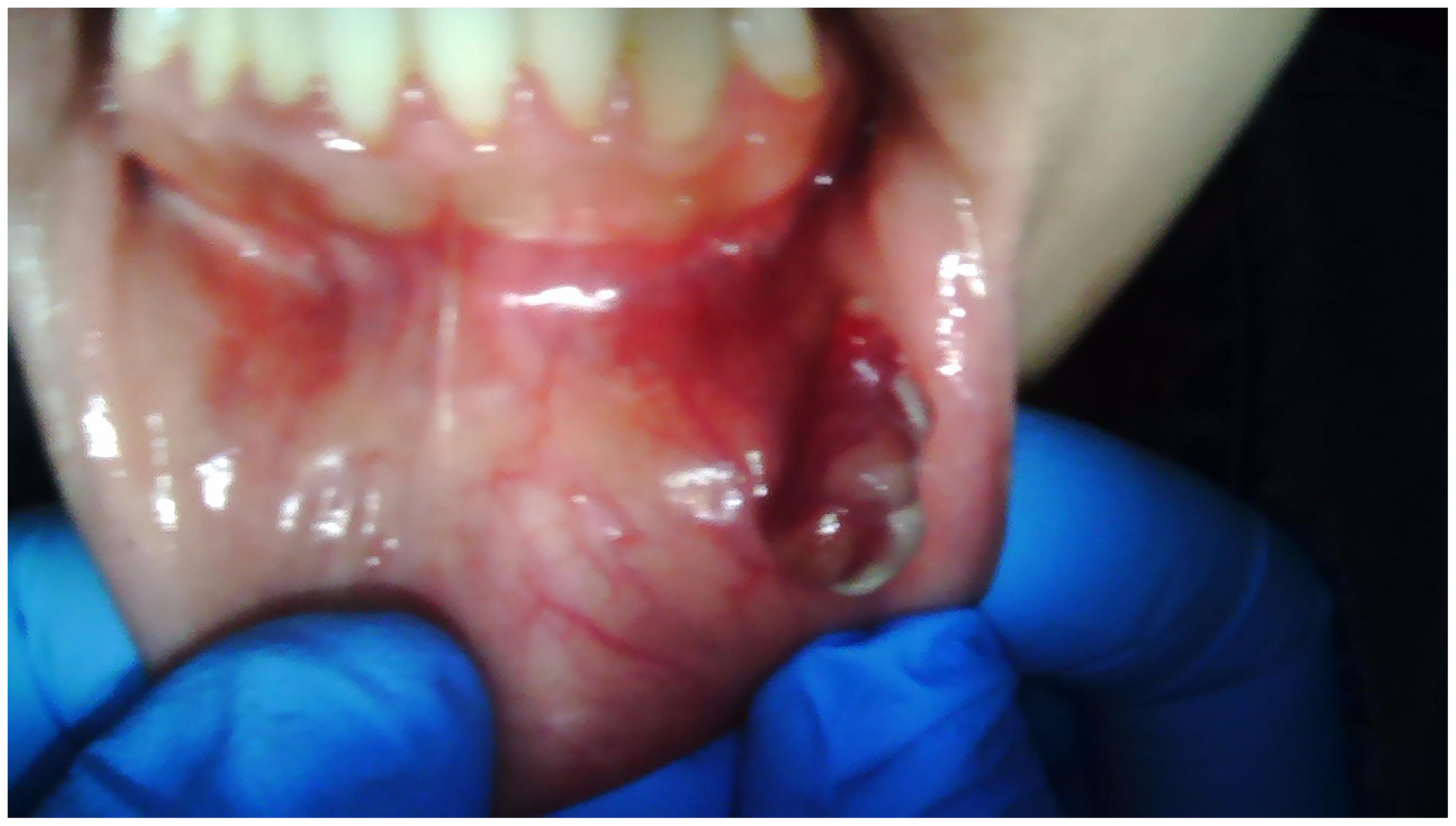
An erythematous, pedunculated nodule was observed on the lower labial mucosa.

Oral examination revealed a dark red, lobulated, pedunculated mass on the lower labial mucosa, measuring approximately 1.0 cm × 0.8 cm (Figure 1). On inspection, the mass had surface ulceration and well-defined margins. On palpation, the mass was soft in consistency, non-tender, and bled readily, with no evidence of induration or fixation to the underlying tissue. General physical examination showed no abnormalities.

Preoperative laboratory tests, including complete blood count, coagulation function, and fasting blood glucose, were all within normal limits. After obtaining informed consent, complete surgical excision was performed under local anesthesia using 2% lidocaine without epinephrine. A fusiform incision was made approximately 1 mm from the lesion margin. The mass and a small amount of surrounding normal mucosa were completely excised, and the wound was sutured. Intraoperative bleeding was minimal. Postoperative care included local wound management without systemic medication. The excised tissue was submitted for histopathological examination.

**Figure 2:**
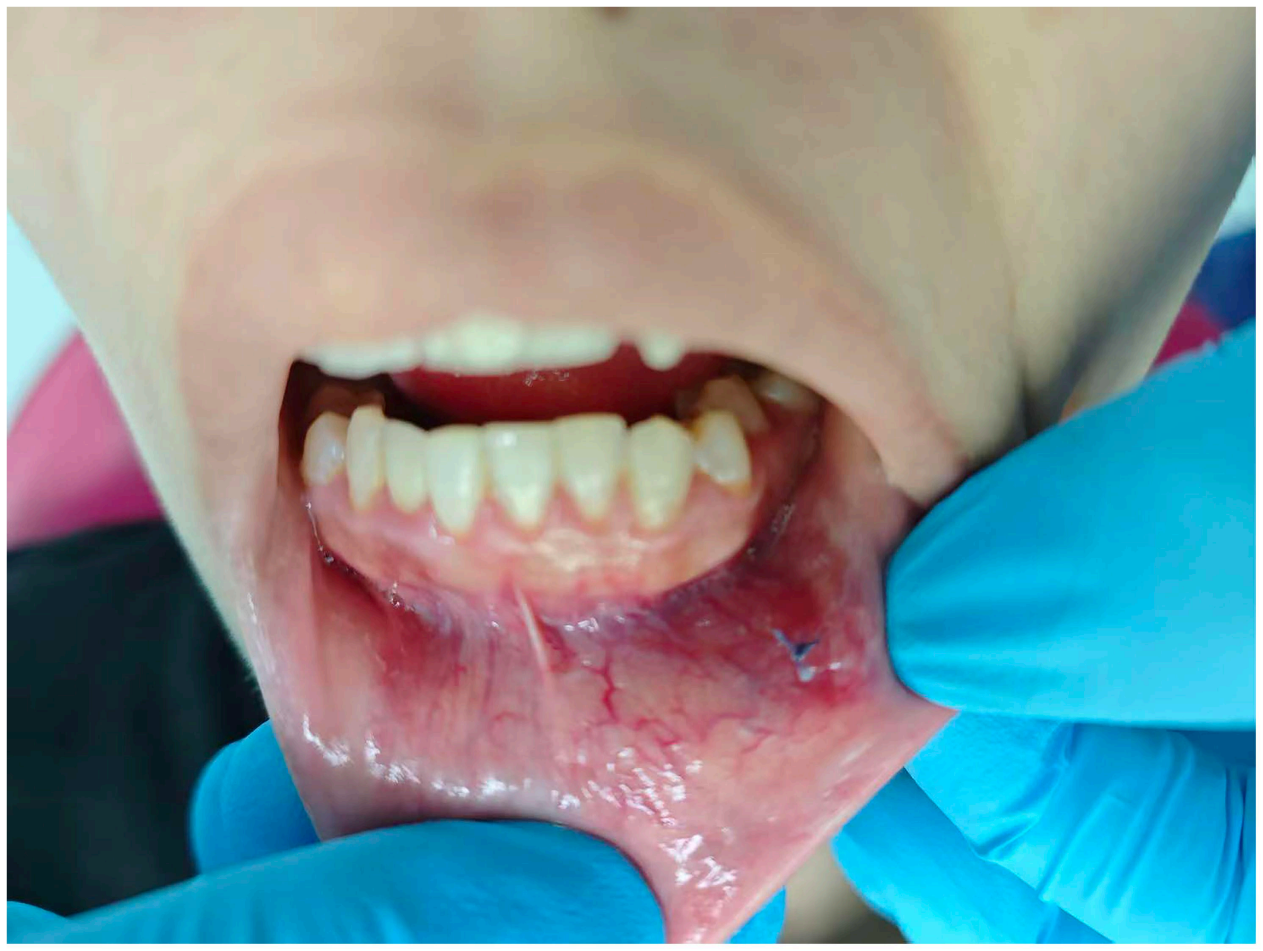
The incision on the lower labial mucosa showed excellent healing.

Sutures were removed 2 weeks postoperatively. The incision healed well, with normal lip appearance and function (Figure 2). At 3-month follow-up, no recurrence was observed, and the patient reported no discomfort. Prenatal check-ups remained normal throughout the follow-up period.

**Figure 3:**
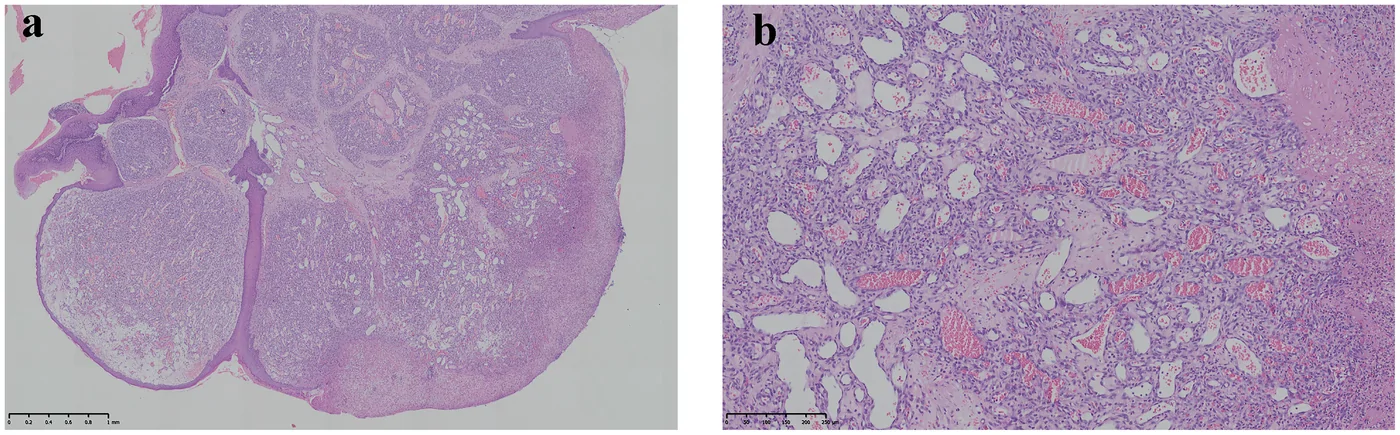
Histopathological findings of the excised lesion. **(a)** Low-power view (H&E, ×2.5) showing lobular proliferation of small vascular channels in the dermis with focal surface ulceration. **(b)** Higher magnification (H&E, ×10) demonstrating vascular endothelial cell proliferation, mixed inflammatory cell infiltrate, and fibrinopurulent exudate on the ulcerated surface.

Gross examination of the specimen revealed a grayish-white, soft tissue mass with a dark red cut surface and no obvious capsule. Microscopic examination showed lobular proliferation of small vascular channels in the dermis, with numerous capillary-sized vessels containing erythrocytes organized in a characteristic lobular pattern (Figure 3a). The vascular endothelial cells demonstrated reactive proliferation without nuclear atypia or mitotic figures. The intervening stroma showed a mixed inflammatory cell infiltrate composed of neutrophils, lymphocytes, and plasma cells. The overlying mucosal epithelium was focally ulcerated and replaced by a fibrinopurulent exudate (Figure 3b). No granulomas, multinucleated giant cells, or areas of necrosis were identified. The histopathological findings were characteristic of lobular capillary hemangioma, consistent with pyogenic granuloma.

### Patient’s perspective

The patient reported relief after surgical excision, as the recurrent bleeding and uncertainty about the nature of the lesion had caused significant anxiety during her pregnancy. She stated that the procedure did not affect her pregnancy course and expressed satisfaction with the functional and aesthetic outcome.

## Discussion

The typical histopathological presentation of PG is lobular capillary hemangioma. For clarity, this report uses “pyogenic granuloma (PG)” for the clinical diagnosis and “lobular capillary hemangioma” for the pathological diagnosis. Oral PG accounts for approximately 1.49%–2.25% of oral biopsy specimens (1, 2). The incidence of this lesion is about 5% during pregnancy, where it is termed “gestational pyogenic granuloma” (3). The gingiva is the most common site for gestational PG (4). The widely accepted mechanism involves local irritation from dental plaque and calculus on the gingival papilla. Additionally, elevated estrogen and progesterone levels during pregnancy enhance the expression of angiogenic factors, such as vascular endothelial growth factor (VEGF) and basic fibroblast growth factor (bFGF). These hormonal changes also inhibit apoptosis in granuloma cells, driving the high incidence of the lesion on the gingiva (5). Although the labial mucosa is another common site for oral PG, gestational cases involving this location are rarely reported. A literature search identified three previously published cases of gestational or pregnancy-associated PG of the labial mucosa or lower lip (6–8). The clinical features, management, and outcomes of these cases are summarized in Table 1 and compared with the present case. Notably, all three previously reported cases presented during the third trimester or postpartum. As a result, none of these reports addressed the clinical decision-making required for intervention during the second trimester. In contrast, the present case presented at 18 weeks of gestation, representing the earliest reported gestational presentation of labial PG requiring active intervention. This raises a distinct clinical question: can surgical excision be safely performed during the second trimester, and if so, what perioperative precautions are necessary to protect fetal safety? No previous report has addressed this question. The present case demonstrates that second-trimester surgical excision under local anesthesia is feasible and safe. It also provides a structured perioperative management framework that can serve as a reference for clinicians facing similar cases.

**Table 1:** Comparison of the present case with previously reported cases of gestational or pregnancy-associated pyogenic granuloma of the labial mucosa or lower lip.

Feature	Present Case	Adeyemi et al. (2011) (6)	Boza & Ovares (2016) (7)	Canivell-Zabaleta et al. (2018) (8)
Age(years)	26	29	19	NR
Gestational age at onset	18 weeks (2rd trimester)	35 weeks (3rd trimester)	3rd trimester	Postpartum
Location	Lower labial mucosa	Lower lip	Lower labial mucosa	Lower lip
Size	1.0 × 0.8 cm	NR	1.0 cm	0.5 cm
Clinical appearance	Dark red, lobulated, pedunculated, ulcerated	NR	Lobulated, pedunculated, erythematous, ulcerated; satellite lesion (2 mm)	Red-raspberry, spherical
Chief complaint	Recurrent bleeding	NR	Recurrence after prior excision; difficulty speaking and eating	Rapid growth; anemia; cancerphobia
Satellite lesion	Absent	NR	Present	Absent
Recurrence before current treatment	No	NR	Yes (8 days after first excision)	No
Treatment timing	During pregnancy (2nd trimester)	During pregnancy	Postpartum	Postpartum
Anesthesia	Local (lidocaine without epinephrine)	NR	Local (2% lidocaine with epinephrine 1:100,000)	Local
Histopathology	Lobular capillary hemangioma with ulceration	NR	Lobular capillary proliferation with endothelial proliferation; chronic inflammatory infiltrate	Granulation tissue with blood vessels, fibroblasts, chronic inflammatory infiltrate
Follow-up	3 months (no recurrence)	NR (no recurrence)	24 months (no recurrence)	12 months (no recurrence)

NR, Not reported.

In this case, the lesion occurred on the lower labial mucosa without clear local irritants such as residual roots or poor restorations. The primary predisposing factors are considered to be: (1) the systemic effect of elevated hormone levels during pregnancy; (2) repetitive mechanical friction from eating and speaking, or minor trauma, which induces reactive hyperplasia of vascular endothelial cells; and (3) the abundant blood supply of the labial mucosa, which provides a favorable microenvironment for vascular proliferation. Compared with gingival PG, PG of the labial mucosa presents unique clinical features (7). The lesion is superficial and prone to external friction, leading to surface ulceration and recurrent bleeding, as was evident in our patient, who presented with repeated bleeding. In addition, the high mobility of the lip tissue subjects the lesion to constant traction, and repeated stimulation can cause rapid growth, which may lead to a misdiagnosis of a malignant lesion. Furthermore, lesions on the lip have a more direct impact on aesthetics and function, resulting in a stronger patient desire for treatment (7). Given these unique clinical features, the differential diagnosis of gestational labial PG requires particular caution (9). The following conditions should be carefully excluded:

1. Pregnancy tumor (epulis gravidarum): This typically occurs on the gingival papilla and is directly associated with the dentition. In this case, the lesion was located on the lower labial mucosa with no relation to the teeth, allowing for clear differentiation.
2. Mucocele: As the most common benign tumor of the lower lip, it presents as a translucent or pale blue cystic lesion with fluctuation. Aspiration yields mucus, and spontaneous bleeding is rare. In contrast, our patient's mass was dark red and bled easily upon touch. Histopathology showed vascular proliferation rather than mucus extravasation.
3. Oral squamous cell carcinoma: Although rare in pregnancy, some ulcerated malignant lesions can present as masses that bleed easily. In this case, the absence of cellular atypia on histopathology ruled out this diagnosis.
4. Vascular malformation: These are typically congenital lesions with a long clinical course, no history of rapid growth, and often yield a positive postural test. Histopathologically, vascular malformations show thin-walled, irregular vascular channels without the lobular architecture characteristic of PG. The present case was a new-onset lesion with rapid growth during pregnancy. Histopathology showed lobular capillary proliferation, which is inconsistent with vascular malformation.
5. Inflammatory granuloma: This usually has a clear infectious trigger, accompanied by pain and purulent discharge. Our patient showed no obvious signs of infection, and histopathology was dominated by vascular proliferation rather than suppurative inflammation.
6. Capillary hemangioma: True capillary hemangiomas are more common in infants and exhibit a natural course of proliferation followed by spontaneous involution. In contrast, our patient's lesion occurred in an adult during pregnancy, and the histopathological findings of lobular capillary proliferation with inflammatory infiltrate were consistent with PG rather than true hemangioma.
7. Peripheral giant cell granuloma: This typically arises from the gingiva or alveolar mucosa and is histologically characterized by multinucleated giant cells in a vascular stroma. The absence of giant cells on histopathology in our case excluded this diagnosis.

The management of gestational PG must balance lesion control with fetal safety. For asymptomatic, small gingival PG, some scholars recommend deferring treatment until after delivery when hormone levels decline, as some lesions may regress spontaneously (10). Non-surgical options, such as Nd:YAG laser therapy, sclerotherapy, and cryotherapy, have also been reported (11). However, these modalities are not recommended during pregnancy due to the lack of established safety data regarding their effects on the fetus. Laser therapy may pose risks related to thermal effects and potential inhalation of plume, sclerotherapy involves the injection of sclerosing agents with unknown placental transfer profiles, and cryotherapy may induce uterine contractions through pain-mediated pathways. Given the absence of pregnancy-specific safety evidence, surgical excision under local anesthesia remains the most well-established and recommended approach for managing gestational PG when intervention is necessary. In the present case, surgical excision during pregnancy was indicated by recurrent bleeding, progressive enlargement, and interference with oral function. Given these clinical circumstances, surgical excision was performed rather than deferring treatment until after delivery. Our patient was at 18 weeks of gestation, which is the second trimester and considered a relatively safe window for non-obstetric surgery (12). We adopted several optimized strategies. Regarding anesthesia, lidocaine without epinephrine was chosen as it is recommended for dental treatment during pregnancy (13). This approach avoids the potential risk of uterine vasoconstriction associated with epinephrine. As for the surgical technique, the excision aimed to ensure complete removal to prevent recurrence. Simultaneously, tissue defect was minimized to reduce the risk of postoperative scar contracture. In terms of postoperative care, no systemic medications were administered to avoid potential fetal risks, and the patient was instructed to avoid lip biting and hard foods to minimize local irritation. Previous studies have shown that the postoperative recurrence rate of oral PG can reach 15% (14), often associated with incomplete excision or persistent local irritation (1). Due to the high mobility of the labial mucosa and continuous stimulation from chewing and friction, there is a theoretically higher risk of recurrence. Therefore, long-term follow-up until after delivery is necessary to observe the lesion's evolution as hormone levels decline.

This report contributes to the understanding of nongingival gestational PG and provides a perioperative management reference for similar cases during the second trimester. This study has the limitation of a short follow-up period, and longer observation is warranted to assess recurrence risk after delivery. Given the current lack of large-sample data, future studies are needed to further define the characteristics and optimal management of labial mucosal PG.

## Conclusion

This report highlights that gestational pyogenic granuloma can affect the labial mucosa, not just the gingiva. Given its nonspecific clinical presentation, histopathological examination is essential for diagnosis. In the second trimester, complete surgical excision is a safe and effective therapeutic strategy.

## Data Availability

The datasets presented in this study can be found in online repositories. The names of the repository/repositories and accession number(s) can be found in the article/Supplementary Material.
